# Angiodrastic Chemokines in Colorectal Cancer: Clinicopathological Correlations

**DOI:** 10.1155/2018/1616973

**Published:** 2018-04-16

**Authors:** George Emmanouil, George Ayiomamitis, Adamantia Zizi-Sermpetzoglou, Maria Tzardi, Andrew Moursellas, Argyro Voumvouraki, Elias Kouroumalis

**Affiliations:** ^1^Gastroenterology Research Laboratory, University of Crete Medical School, Heraklion, Greece; ^2^Department of Surgery, Tzanion General Hospital, Piraeus, Greece; ^3^Department of Pathology, Tzanion General Hospital, Piraeus, Greece; ^4^Department of Pathology, University of Crete Medical School, Heraklion, Crete, Greece; ^5^Department of Gastroenterology & Hepatology, University Hospital of Heraklion, Heraklion, Crete, Greece

## Abstract

**Aim:**

To study the expression of angiodrastic chemokines in colorectal tumors and correlate findings with clinicopathological parameters and survival.

**Methods:**

The proangiogenic factor VEGF, the angiogenic chemokines CXCL8 and CXCL6, and the angiostatic chemokine CXCL4 were measured by ELISA in tumor and normal tissue of 35 stage II and III patients and correlated with the histopathology markers Ki67, p53, p21, bcl2, EGFR, and MLH1 and 5-year survival. The Wilcoxon and chi-square tests were used for statistical comparisons.

**Results:**

There was a significant increase of CXCL6 (*p* = 0.005) and VEGF (*p* = 0.003) in cancerous tissue compared to normal. Patients with lower levels of CXCL8 and CXCL4 lived significantly longer. Patients with loss of EGFR expression had higher levels of CXCL8 while p21 loss was associated with higher levels of CXCL6. Chemokine levels were not correlated with TNM or Dukes classification. Strong expression of p53 was accompanied by decreased survival.

**Conclusions:**

(1) The angiogenic factors CXCL6 and VEGF are increased in colorectal cancer tissue with no association with the clinical stage of the disease or survival. (2) However, increased levels of tissue CXCL8 and CXCL4 are associated with poor survival. (3) Strong expression of p53 is found in patients with poor survival.

## 1. Introduction

The incidence of cancer is increasing every year. Colorectal cancer (CRC) is the second most common cause of cancer mortality in the Western world [1]. Many factors both environmental and genetic are implicated in the propagation and mortality caused by CRC. Among various trophic factors, chemokines have a predominant role.

Chemokines were originally considered to participate in the chemoattraction of leukocytes to inflammatory sites. Later, it became clear that chemokines and their receptors may also modulate tumor behavior through regulation of angiogenesis, activation of tumor cell proliferation, and metastasis [2].

There are several chemokine families. The CXC chemokine family is particularly implicated in the modulation of different cancers.

CXC chemokines are subdivided into ELR^−^ and ELR^+^ subgroups based on the presence or absence of the ELR motif glu-leu-arg. ELR^+^ chemokines (CXCL1, 2, 3, 5, 6, 7, and 8) are angiogenic factors, whereas ELR^−^ members are mostly angiostatic factors and inhibit the formation of new blood vessels that are critical for tumor expansion [3].

*CXCL8 (IL-8)* bears the ELR motif and is the most potent human neutrophil chemoattractant and activator [4]. CXCL8 is the first chemokine to be reported as an angiogenic factor [5]. Several studies describe an upregulation of CXCL8 in colon cancer cells and surrounding stromal cells [6–8] under the influence of various proinflammatory cytokines, such as IL-1*β* and TNF-*α*, and even microorganisms and hypoxia [9–13].

Several effects of CXCL8 favor the progression of colorectal adenocarcinoma. Thus, it induces transendothelial neutrophil migration and increases the expression of intercellular adhesion molecule-1 (ICAM-1) on colon cancer cells resulting in extensive leukocyte adhesion to these cells [11]. There have been also reports that it mediates the binding of colon carcinoma cells to endothelial cells, which favors tumor cell invasion and metastasis [14], and importantly, it also promotes the outgrowth of newly formed vascular vessels thus providing critical oxygen and nutrients to the tumor [5, 15–17]. In addition, it acts as an autocrine growth factor for colon adenocarcinoma cells [14, 16–18].

*CXCL6 (GCP2)* is also an ELR^+^ CXC chemokine sharing 31% amino acid sequence homology with CXCL8 and similar properties. It stimulates the secretion of proteases such as matrix metalloproteinase-9 (MMP-9) from the granules of granulocytes [19–22]. CXCL6, like CXCL8, binds to the CXCR1 and CXCR2 receptors, which mediate their chemotactic and angiogenic activities [23–25].

*VEGF* is a strong angiogenic factor important for tumor neovascularization. Binding to three structurally similar receptors leads to endothelial cell proliferation, migration, survival, and angiogenesis [26, 27] which is further supported by extravasation of plasma proteins into the extravascular space, clotting formation, and deposition of fibrin that serves as matrix for the growth of new blood vessels and mesenchymal cells [28]. VEGF also attracts macrophages that may influence tumor progression [29].

*CXCL4* is an ELR^−^ CXC chemokine. Therefore, it is angiostatic and also inhibits endothelial cell chemotaxis [5, 30, 31]. CXCL4 is the first angiostatic chemokine described and has been shown to inhibit the angiogenic effects of VEGF and bFGF [32, 33]. There are two CXCL4 variants (CXCL4 and CXCL4L1) both with angiostatic properties, although CXCL4L1 is considered a more potent inhibitor of angiogenesis, differing only by three amino acids [31]. CXCL4 binds to the CXCR3 receptor [34, 35] but also inhibits angiogenesis via interaction with cell surface glycosaminoglycans or with angiogenic mediators and their receptors such as bFGF and CXL8 [36–38].

There have been earlier studies on the expression of mostly angiogenic factors in colorectal carcinoma, but simultaneous studies of angiostatic and angiogenic chemokines are missing. We studied therefore the expression of two relatively less well-studied chemokines CXCL6 and CXCL4 along with the better-studied CXCL8 (IL-8) and VEGF in both carcinoma and adjacent noncancerous tissue and correlated with several cancer indices, trophic factors, and patient survival.

## 2. Patients

Patients with biopsy-confirmed colorectal cancers were recruited for participation in the current study. The study was conducted in accordance with the Declaration of Helsinki and was approved by the Ethics Committee of the University Hospital of Heraklion, Heraklion. In all cases, written consent from the participants was obtained. Selection of patients was based on the following criteria:

- Only patients with stages II and III according to TNM classification (stages B and C according to Dukes classification as modified by Astler-Coller) were included. Metastatic disease (type IV) patients were excluded [39–41].
- Only patients with a curative (R0) surgical resection who did not receive adjuvant chemotherapy (either refused or were not considered as candidates by the attending doctors) were included.
- Only patients with at least a 5-year follow-up (or death before that) were included in the report. Patients lost to follow-up were not included.

In all, 35 patients operated for left colorectal adenocarcinoma fulfilled the criteria and were included in the study. Patient demographics are presented in Table 1.

At operation, once the tumor was resected, two tissue samples were collected, one from the tumor itself and a second from apparently normal mucosa about 10 cm away from the resection margin. Both samples were immediately frozen in liquid nitrogen and then stored at −80°C until studied. A standard pathological evaluation was done with the rest of the tumor specimen.

## 3. Pathology

For every patient, immunohistochemical detection of MLH1, Ki-67, bcl2, p53, and p21 and EGFR protein expression were studied in tumor tissue by immunoperoxidase staining in 3 steps using a Dako kit as previously described [42].

Primary antibodies were antihuman MutL protein homologue-1 (MHL1), clone E 505 (ready for use, Dako), anti-Ki-67 (MIB-1Ab, dilution 1 : 80, Dako), anti-p53 (DO-7, dilution 1 : 100, BioGenex), anti-p21 (dilution 1 : 40, Dako), anti-bcl2 (dilution 1 : 10, BioGenex), and anti-EGFR (dilution 1 : 40, Dako). Tumors with known Ki-67, p53, and p21 and EGFR status were used as positive controls, whereas a normal lymph node served the same purpose for bcl2.

Ki-67, MLH1, bcl2, p53, and P21 expression was scored as previously described by two pathologists without knowledge of the clinical details [42] and according to previous studies [43–45]. EGFR expression was assessed according to the percentage of positive cells using the “0 to 2+” scale as follows: a score of 0 is an absence of positive cells; a score of 1 is >1–3%; and a score of 2+ is >4% positive cells.

## 4. Materials and Methods

Concentration of 4 chemokines (CXCL8, CXCL4, CXCL6, and VEGF) was calculated through ELISA protocols. All tissue samples, both cancer and control, were homogenized with a glass homogenizer in 1 ml 0.25% BSA phosphate-buffered saline (PBS) on ice. Immediately after homogenization, the samples were aliquoted and frozen at −80°C till further analysis. For each one of these chemokines, commercially available monoclonal antibodies and biotinylated antibodies were obtained (R&D Systems).

These antibodies were reconstituted with sterile Tris-buffered saline (TBS) according to the manufacturer's instructions. After reconstitution, antibodies were aliquoted and stored at −20°C. Apart from the antibodies, commercially available recombinant human CXCL8, CXCL4, CXCL6, and VEGF were obtained (R&D Systems) to create solutions of known concentration for generating standard curves. Every sample was run in duplicate. Incubation times and antibodies' concentrations were set in each protocol according to the manufacturer's instructions.

Total tissue protein concentration was calculated in each homogenized sample using the bicinchoninic acid (BCA) method. Optical absorption was measured at 540 nm, and protein concentration was calculated through a standard curve with standards of known protein concentration. This method was preferred for our study as it can produce more accurate results in samples with high total protein concentration [46]. Chemokine concentrations were expressed as pg per ng of total protein.

## 5. Statistics

Statistical analysis was performed with the IBM SPSS statistics software version 19. Results are expressed as means ± standard deviation of the mean and were depicted as box plots. The nonparametrical test Wilcoxon signed-rank test for paired samples was used when the Kolmogorov-Smirnov method showed that the distribution of values was not normal. The chi-square test for the analysis of nonparametric data in 2 × 2 tables was used for associations between histopathology markers and 5-year survival. Statistical significance was set at the 5% level (*p* = 0.05).

## 6. Results

**Figure 1:**
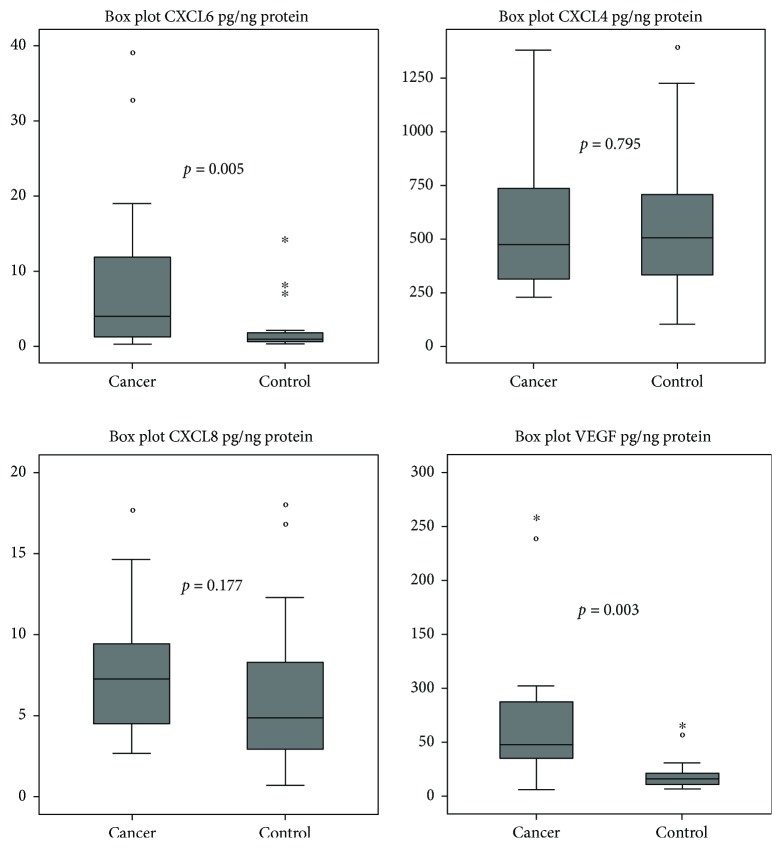


The Kolmogorov-Smirnov test showed that chemokine concentrations in both tumor and normal tissues were not normally distributed.  Figure 1 shows that there are no significant differences between tumor and normal tissue for CXCL8 (*p* = 0.177) and CXCL4 (*p* = 0.795).

However, there was a significant difference for CXCL6 (*p* = 0.005) and VEGF (*p* = 0.003) between tumor and normal tissue.

## 7. 5-Year Survival

Due to lack of normality in distribution, the Wilcoxon test was used for comparisons. As shown in Table 2, there is a significant difference in 5-year survival for CXCL8 and CXCL4. Patients who survived had significantly lower levels of those chemokines as compared to nonsurvivors. The same tendency existed for CXCL6 and VEGF, but this was not statistically significant due to the high scattering of results.

Table 3 shows that increased expression of p53 is associated with a significantly reduced five-year survival, while patients with no expression of p21 and MLH1 tend to live longer, but this tendency was not significant statistically, possibly due to the relative small number of patients. Increased expression of EGFR was also associated with increased survival, but this also was not statistically significant.

## 8. Dukes Staging and TNM Staging

No difference of chemokine levels was found according to Dukes or TNM staging (Tables 4 and 5).

## 9. Histopathology Markers

### 9.1. Ki67, Bcl2, p53, and MLH1

Table 6 shows that there is no statistical difference between chemokine levels and expression of these markers. However, reduced expression of Ki67, p53, and MLH1 was associated with high, although nonsignificant, levels of CXCL6.

### 9.2. EGFR

Lack of expression of EGFR was associated with almost twice as high levels of CXCL8. On the other hand, no expression of EGFR was associated with reduced levels of CXCL6, but this was not significant due to high scattering of results. Results are shown in Table 7.

### 9.3. p21

As shown in Table 8, lack of expression of p21 is associated with significantly increased levels of CXCL6. By contrast, expression of p21 is associated with higher levels of VEGF, but this was not significantly different due to scattering of results.

## 10. Discussion

It is usually stated that bowel adenocarcinomas arise from epithelial cells. However, it is accepted today that the interaction between tumor cells and the tumor microenvironment is equally important for tumor evolution. Among others, expression of angiodrastic agents is particularly effective as they can induce or inhibit neovascularization, a process vital for tumor progression [47, 48]. Colorectal cancer is the second leading etiology of cancer death in Western countries. Almost half of the patients die of metastatic disease after curative surgery despite adjunct chemotherapy [49, 50]. TNM classification is considered to be the best prognostic factor in early stages of colon cancer [51].

In the present study, we measured in tumor tissue the quantity of the angiogenic chemokines CXCL6 and CXCL8 (IL-8) and the most widely studied angiogenic factor VEGF along with the angiostatic chemokine CXCL4. For comparisons, the same angiodrastic chemokines were assessed in normal colonic tissue from the same patients.

Chemokines are implicated in cancer tumorigenesis and metastasis affecting tumor microenvironment mainly through development of local inflammation and angiogenesis [52].

CXC chemokines also attract neutrophils and lymphocytes thus modulating innate and adaptive immunity and interfering apoptosis, proliferation, and tumor cell metastases. Invasion and metastasis are dependent on a proangiogenic environment [53].

We have demonstrated that levels of two angiogenic factors, the chemokines CXCL6 and VEGF, are significantly higher in the malignant tissue compared with those in the normal tissue. Moreover, increased levels of two other chemokines the proangiogenic CXCL8 (IL-8) and the angiostatic CXCL4 are associated with a worse 5-year survival. Our findings for tissue levels of CXCL8 are different from those reported in the literature as we failed to demonstrate increased CXCL8 levels in the tumor tissue compared with those in the adjacent normal bowel. This is in disagreement with several reports that CXCL8 is upregulated in colon cancer cells and surrounding stromal cells in comparison with its normal counterparts [6–8, 54]. This is further supported in a recent study where CXCL8 expression was significantly upregulated in tumoral samples compared with that in normal tissue, and this upregulation increased with patients' age [55]. The explanation for this discrepancy might be found in the report by Ning et al., where patients with stage IV CRC had more than 10 times higher serum level of CXCL8 compared with individuals with no evidence of disease [17]. Unlike other reports, we included only patients with disease stages II and III to avoid interference in survival by already present dissemination of the tumor, and this exclusion of stage IV patients where the highest values of CXCL4 are observed may explain our findings.

In an effort to see if chemokine levels are associated with survival and TNM or Dukes classification, we studied only patients who did not receive any adjunct chemotherapy after curative resection. In our study group, the overall 5-year survival was 55.1% similar to the previous study reporting stage-specific survival rates of 96%, 87%, 55%, and 5% for TNM stages I, II, III, and IV, respectively [56]. Interestingly, poor survival was related to increased CXCL8 and CXCL4 levels. The same tendency of increased levels in nonsurvivors also existed for VEGF, but this was not statistically significant. Our findings of CXCL8 and 5-year mortality are in agreement with previous reports where reduced overall survival in colorectal tumors is associated with high levels of CXCL8 [57–59]. In a large recent meta-analysis, increased levels of CXCL8 were associated with poor prognosis, but this was evident mostly in stage IV TNM patients while the association was weaker with overall survival [60]. This association is obviously due to the profound trophic effect of CXCL8 on human colon cancer cells along with increased peritumoral neoangiogenesis and extravasation of tumor cells into the liver and lung [61]. The detrimental effect of CXCL8 is further supported by its upregulation of MMPs from tumor cells hence increasing their potential for metastasis [62].

On the other hand, the association of CXCL8 with mortality may be related to the origin of CXCL8. An increased expression of CXCL8 in the peritumoral inflammatory infiltrate was associated with improved disease-free survival [63]. This is possibly due to the effect of CXCL8 on neutrophil recruitment. The proangiogenic effects of CXCL8 are independent from its chemotactic activity for neutrophils and other proinflammatory effects [64]. Neutrophils, attracted by CXCL8, might affect tumor development in two discrete ways depending on their phenotype. N1 tumor-associated neutrophils (TAN) contribute to tumor immune surveillance due to their cytotoxic ability and interaction [65].

Despite the fact that VEGF was significantly increased in tumor tissue compared with that in the adjacent normal tissue in our patients, there were no significant differences between VEGF levels and either histological classification (TNM and Dukes) or more importantly with patient survival. There are two possible explanations for this. First, the role of angiogenesis as a prognostic factor is still controversial. Some other studies pointed out that measurements of angiogenesis do not provide relevant prognostic information [66–68]. Moreover, an interaction of VEGF with CXCL8 may be more important than the expression of either factor alone. There is evidence that activated neutrophils generate VEGF which in turn induces upregulation of the antiapoptotic protein bcl-2 in endothelial cells that promotes the expression of endothelial cell production of CXCL8 [69, 70]. It should be noted however that levels of VEGF in our study were also increased in nonsurvivors, but this was not statistically significant. High levels of VEGF expression have been associated with advanced cancer stage and related with unfavorable prognosis [71–73]. However, a recent publication may offer a more sound explanation. It was reported that the expression of CXCL4 in colon cancer seems to counterbalance the angiogenic effects of both VEGF and CXCL8. It is therefore possible that it is the relative expression of different chemokines and the resultant chemokine environment that influence the potential progression of colorectal tumors [74]. This may account for the association of the angiostatic CXCL4 with survival in our patients. It is plausible to assume that it is the balance of angiogenic and angiostatic factors that influences the end result in survival. We found increased levels of CXCL4 associated with decreased survival, but it may be postulated that these levels could not counteract the detrimental effect of CXCL8 and VEGF.

Data on CXCL6 in colorectal cancer are very limited. We found significantly increased levels of CXCL6 in tumor samples compared with those in the adjacent normal tissue. This is in agreement with the findings of Gijsbers et al. [75] who detected CXCL6 in endothelial cells of colorectal adenocarcinomas, but not in endothelial cells of normal tissues.

The production of CXCL6 by endothelial cells within the tumor would imply that it might interfere with tumor development, invasion, and metastasis through neovascularization, but this is not substantiated by our findings since no differences were found in CXCL6 levels according to either TNM or Dukes classification. These are in contrast to other findings where no difference in either mRNA expression or protein concentration of CXCL6 between cancer and normal tissues was found probably due to the different patients studied [76].

Another interesting finding of our study is the lack of association of traditional histopathology markers with survival. Only patients with a strong expression of p53 had significantly decreased survival. It should be noted however that there was a trend for increased survival in patients with reduced expression of MLH1 and p21 and increased expression of EGFR, but this was not statistically significant.

Results from previous reports on the association of histopathology markers with survival are contradictory. In accordance with our study, the expression of bcl2 was not correlated with neither angiogenesis nor survival in Greek patients [77]. Similarly, only young patients, less than 40 years, had worst prognosis when tumors were bcl2 negative [78]. However, two other studies reported that bcl2 expression was associated with increased survival [79, 80]. Similarly, lack of bcl-2 expression was correlated with increased relapses while bcl-2 immunodetection was accompanied by slower local tumor growth [81]. Considering p53 expression, we found significantly decreased survival of patients with strong expression of p53, in accordance with previous reports [79, 82].

P21 is a cyclin-dependent kinase inhibitor that controls cell cycle arrest. Upregulation of p21 inhibits cell growth and silencing favors tumor proliferation [83].

Previous data on p21 loss and clinical outcome in colon cancer have not been conclusive. While p21 loss has been associated with poor prognosis [84], most studies showed no independent prognostic value of p21 [85–87]. P21 loss in colon cancer was reported to be associated with longer survival among patients ≥ 60 years old, whereas it is associated with shorter survival among patients < 60 years old [88]. Our patients with no expression of p21 tend to live longer, but this tendency was not significant statistically possibly due to the relative small number of patients.

The expression of EGFR seems to be dependent on the site of tumor development. EGFR was positive in 92% of 619 tumor samples in a large series of colorectal tumors and EGFR expression correlated with favorable survival [89]. Another study of more than 10,000 tumors reported that EGFR expression was identified in approximately 45% of the left colon tumors, a finding similar to ours where 47% of patients were expressing EGFR [90]. In our study, increased expression of EGFR was also associated with increased survival, but this was not statistically significant. Interestingly, lack of expression of EGFR was accompanied by significantly higher levels of CXCL8 (Table 7). This is in agreement with our finding that increased levels of tumor CXCL8 are associated with decreased survival. An explanation for this might be the reported direct stimulation of cancerous cell proliferation by upregulation of EGFR and by proteolytic processing of EGFR ligands mediated by CXCL8 [91].

Microsatellite instability (MSI) is a form of genetic instability caused by alterations in the DNA mismatch repair system. MSI is due to a germline mutation in one of the mismatch repair genes (MLH1, MSH2, MSH6, and PMS2) or to epigenetic silencing of MLH1.

Methylation of the MLH1 promoter mediates gene silencing and leads to a reduction or loss of MLH1 expression. Loss of MLH1 expression is considered to be a rapid and reliable test in identifying the MSI-H (high) phenotype of colorectal cancers [92, 93]. However, there is some confusion regarding mortality and MLH1 expression. Thus, loss of MLH1 expression was detected in approximately 90% of MSI-H carcinomas. Patients with MLH-1-negative carcinomas had increased mortality compared with those patients with MLH-1-positive tumors, but this was not significantly different [94]. On the other hand, MSI-H colorectal tumors have been associated with longer survival, better prognosis, and less tendency to metastasize than stage-matched tumors with microsatellite stability [95–98]. These reports are in agreement with our results. Our MLH1-negative patients tend to have a better 5-year survival, but as in the case of p21 expression, this was not statistically significant.

In conclusion, our results showed that the concentration of two angiogenic factors VEGF and CXCL6 is significantly increased in colorectal tumor tissue as compared with that in the adjacent normal tissue; therefore, they might be involved in local angiogenesis and tumor expansion. Moreover, significantly increased CXCL8 and CXCL4 levels were associated with a worse 5-year survival. The same nonsignificant trend was observed for VEGF. Chemokine levels were not related to histological tumor classification, but tumors with no expression of EGFR and p21 had significantly increased levels of CXCL8 and CXCL6, respectively.

## Figures and Tables

**Table 1:** Patients' demographics.

Characteristics	Number
Sex	
Male	20
Female	15
Smoking	
Yes	21
No	14
BMI	
<25	15
>25	20
Dukes staging	
B	16
C	19
TNM	
II	16
III	19
Tumor location	
Rectum	16
Sigmoid	15
Left colon	4

**Table 2:** 5-year survival of patients according to chemokine levels. Wilcoxon signed-rank test for paired samples.

Chemokine	5-year survival	Mean (pg/ng protein)	Std deviation	*p* value
CXCL8 (cancer)	No	8.17	3.82	0.028
	Yes	4.45	1.70	
CXCL6 (cancer)	No	15.13	19.17	0.841
	Yes	5.78	4.97	
VEGF (cancer)	No	101.33	96.37	0.306
	Yes	45.34	25.30	
CXCL4 (cancer)	No	603.03	307.42	0.028
	Yes	323.79	120.73	

**Table 3:** 5-year survival of patients according to histopathology expression. Chi-square test.

Biomarker	Intensity scores	5-year % alive	*p* value
Ki67	1-2	33.3%	1.000
	3	33.3%	
p53	0–2	57.1%	0.036
	3	12.5%	
p21	0	50.0%	0.264
	1–3	22.2%	
Bcl2	0	33.3%	1.000
	1	33.3%	
MLH1	0	50.0%	0.264
	1	22.2%	
EGFR	0	16.7%	0.264
	1-2	44.4%	

**Table 4:** Chemokine levels according to Dukes classification. Wilcoxon signed-rank test for paired samples.

Chemokine	Dukes staging	Mean (pg/ng protein)	Std deviation	*p* value
CXCL8 (cancer)	B	8.20	4.53	0.773
	C	7.54	4.30	
CXCL6 (cancer)	B	9.22	11.56	1.000
	C	8.69	12.23	
VEGF (cancer)	B	78.54	78.15	0.834
	C	70.71	72.20	
CXCL4 (cancer)	B	577.81	365.63	0.773
	C	574.45	333.62	

**Table 5:** Chemokine levels according to TNM staging. Wilcoxon signed-rank test for paired samples.

Chemokine	TNM staging	Mean (pg/ng protein)	*p* value
CXCL8 (cancer)	II	7.73	0.773
	III	7.98	
CXCL6 (cancer)	II	9.44	0.773
	III	8.38	
VEGF (cancer)	II	70.51	0.560
	III	79.91	
CXCL4 (cancer)	II	548.02	0.501
	III	607.55	

**Table 6:** Chemokine levels according to histopathology marker expression. Wilcoxon signed-rank test for paired samples.

	Intensity scores	CXCL8 (cancer)			CXCL6 (cancer)			VEGF (cancer)			CXCL4 (cancer)		
		Mean (pg/ng protein)	SD		Mean (pg/ng protein)	SD		Mean (pg/ng protein)	SD		Mean (pg/ng protein)	SD	
Ki67	*1-2*	7.97	3.43	NS	11.06	14.66	NS	104.62	101.73	NS	486.85	170.26	NS
	*3*	7.77	4.97		7.78	10.09		51.3	27.81		638.46	416.03	

Bcl2	−ve	7.6	3.83	NS	9.18	12.59	NS	88.76	79.16	NS	553.72	299.45	NS
	+ve	8.65	6.17		8.15	8.68		32.22	17.95		648.55	490.81	

p53	−ve	5.93	2.81	NS	11.79	15.46	NS	80.71	75.95	NS	506.56	249.26	NS
	+ve	9.56	4.77		6.4	6.49		68.54	74.17		637.79	405.96	

MLH-1	−ve	8.89	5.35	NS	11.18	12.92	NS	102.39	102.61	NS	588.84	391.89	NS
	+ve	7.12	3.47		7.72	11.19		53.03	28.96		567.07	316.73	

**Table 7:** EGFR expression and chemokines. Wilcoxon signed-rank test for paired samples.

Chemokine	EGFR intensity scores	Mean (pg/ng protein)	Std deviation	*p* value
CXCL8 (cancer)	0	10.17	3.91	0.012
	1-2	5.78	3.61	
CXCL6 (cancer)	0	5.99	6.37	0.847
	1-2	11.56	14.65	
VEGF (cancer)	0	77.89	65.52	0.368
	1-2	70.44	86.52	
CXCL4 (cancer)	0	672.37	313.01	0.124
	1-2	490.40	353.59	

**Table 8:** Chemokine levels and p21 expression. Wilcoxon signed-rank test for paired samples.

Chemokine	p21	Mean (pg/ng protein)	Std deviation	*p* value
CXCL8 (cancer)	−ve	7.52	4.71	0.501
	+ve	8.14	4.13	
CXCL6 (cancer)	−ve	14.73	14.27	0.016
	+ve	3.79	4.99	
VEGF (cancer)	−ve	45.17	29.89	0.208
	+ve	104.08	92.08	
CXCL4 (cancer)	−ve	579.20	389.80	0.773
	+ve	573.22	308.73	
